# Supplementary material for: Fusobacterium nucleatum promotes tumor extravasation and metastasis in head and neck cancer via TLR4/MYB/ESPN axis
Source: Commun Biol. 2026 Mar 25;9:664. doi: 10.1038/s42003-026-09913-3 (PMC13181126; doi:10.1038/s42003-026-09913-3)

SUPPLEMENTARY INFORMATION

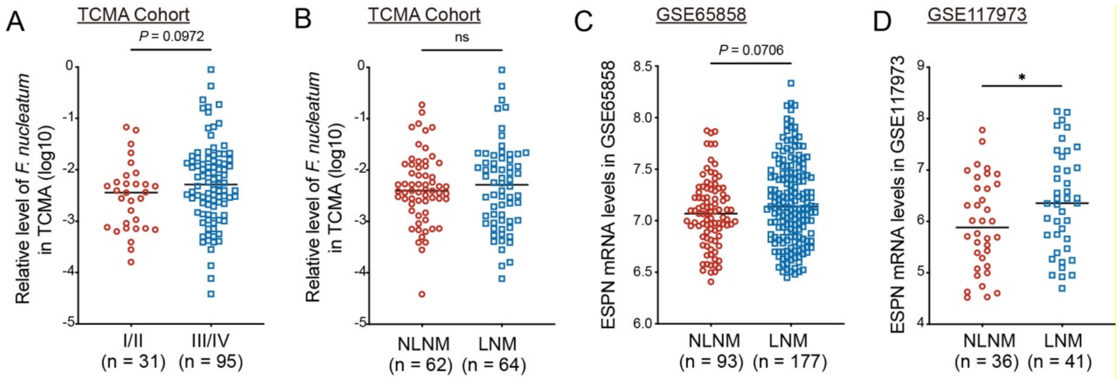

Supplementary Figure S1. Validation of the association of *F. nucleatum* abundance and ESPN expression with lymph node metastasis using public databases. (**A**) Relative abundance of *F. nucleatum* in patients with HNSCC with or without lymph node metastasis (LNM) in TCMA cohort (n = 126, samples without detectable *F. nucleatum* were excluded). (**B**) Relative abundance of *F. nucleatum* in patients with HNSCC across different TNM stages in TCMA cohort. (**C**) ESPN mRNA levels in tumor tissues with or without LNM in GSE65858 cohort (n = 270). (**D**) ESPN mRNA levels in tumor tissues with or without LNM in GSE6117973 cohort (n = 77). Bars indicate mean with SD (A, B, D), and median with interquartile range (C). ns, nonsignificant; * $P < 0.05$. LNM, lymph node metastasis. NLNM, no lymph node metastasis.

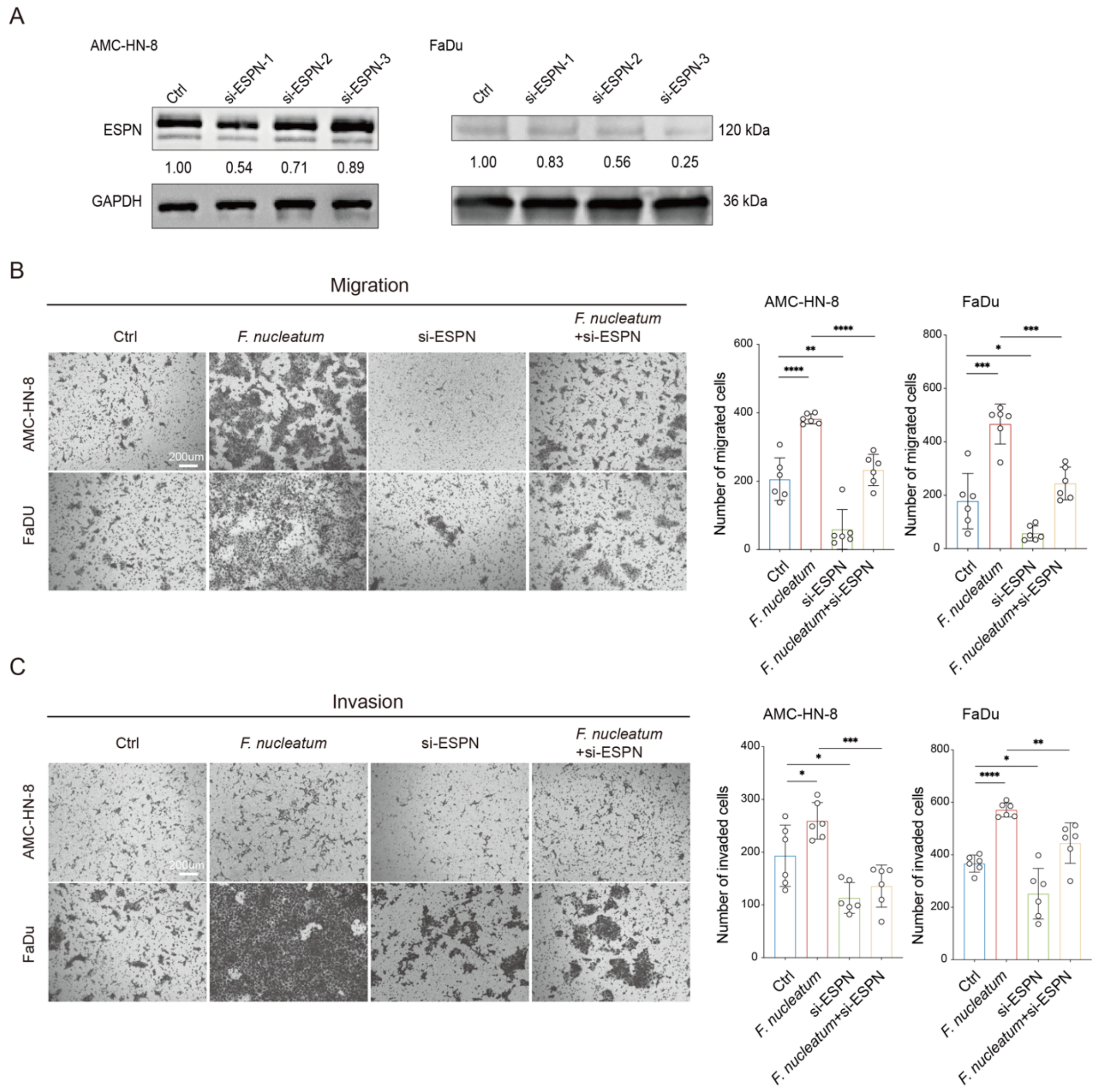

Supplementary Figure S2. *F. nucleatum* promotes HNSCC cells extravasation by upregulating ESPN protein, related to Figure 3. (**A**) Western blot analysis showing the siRNA interfering efficiency. (**B** and **C**) Transwell assays showing the migratory (B) and invasive (C) abilities of *F. nucleatum*-treated HNSCC cells pretreated with ESPN siRNA (n = 6).

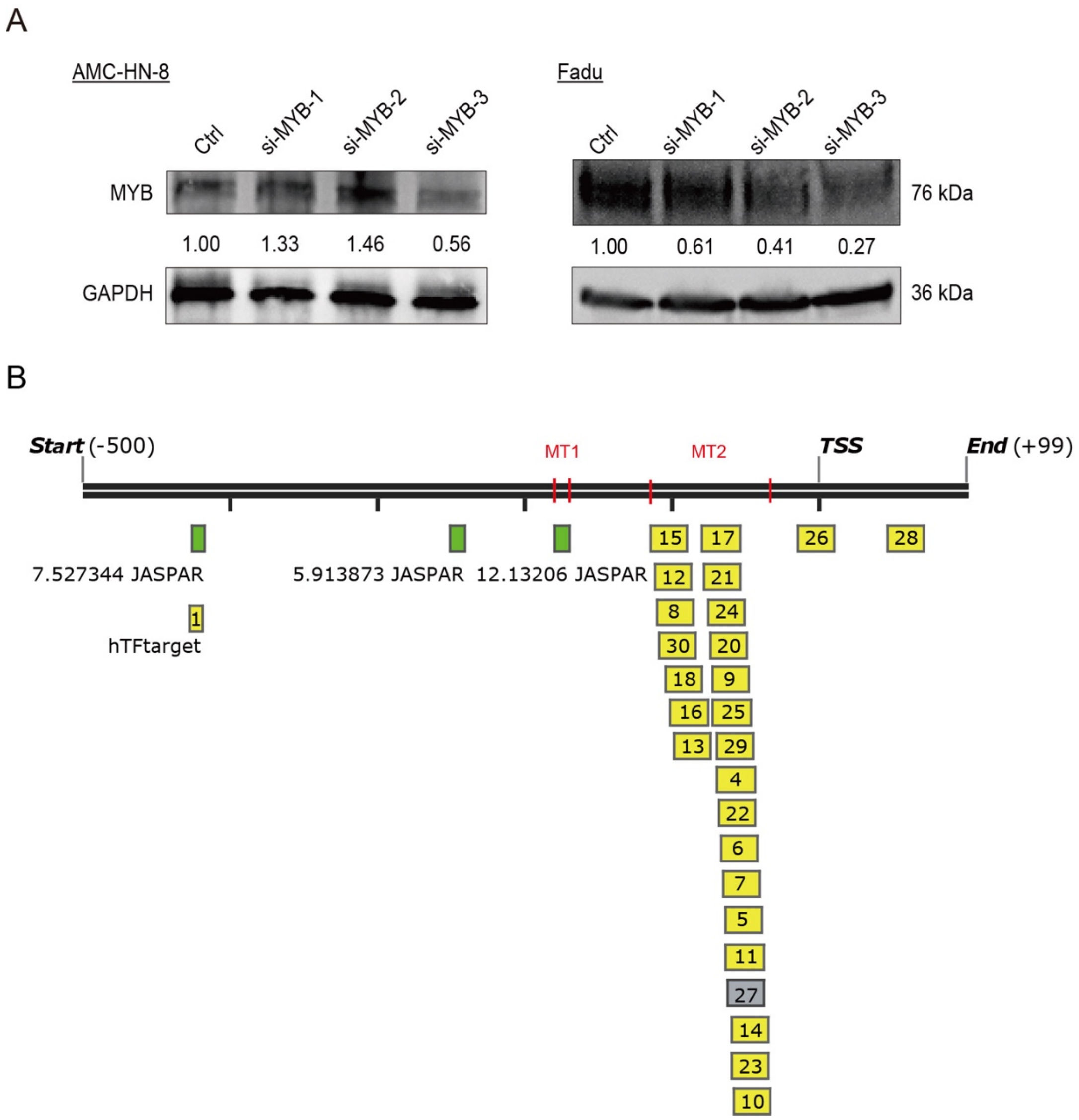

Supplementary Figure S3. *F. nucleatum* upregulates MYB to elicit ESPN transcription, related to Figure 4. (**A**) Western blot analysis showing the siRNA interfering efficiency. (**B**) Schematic representation of predicted MYB binding sites within the P3 fragment (-500 to +99) of the ESPN promoter. Potential MYB binding sites were predicted using JASPAR and hTFtarget databases, the highest-scoring site (MT1) was selected from the JASPAR database, while the most frequently repeated site (MT2) was chosen from the hTFtarget database.

Supplementary Table S1. Predicted MYB binding sites within the fragment (-500 to +99) of the ESPN promoter in the JASPAR databases

Matrix ID	Name	Score	Start	End	Predicted sequence
MA0100.3	MA0100.3.MYB	12.1321	1822	1831	ctcaactgtt
MA0100.3	MA0100.3.MYB	7.52734	1575	1584	gacaacggcc
MA0100.3	MA0100.3.MYB	5.91387	1751	1760	ttcatctgcc
MA0100.3	MA0100.3.MYB	5.42449	107	116	tgctactgct
MA0100.3	MA0100.3.MYB	5.41747	528	537	cccaacagta
MA0100.3	MA0100.3.MYB	5.21991	1189	1198	ctccactgaa
MA0100.3	MA0100.3.MYB	5.18066	920	929	tcccactgtt
MA0100.3	MA0100.3.MYB	5.16327	563	572	cacacctgcc
MA0100.3	MA0100.3.MYB	5.08426	17	26	tacaactatt
MA0100.3	MA0100.3.MYB	5.0747	310	319	tgccactgtt
MA0100.3	MA0100.3.MYB	5.01745	1468	1477	gagaactgcc
MA0100.3	MA0100.3.MYB	4.96514	330	339	ggcatctgct
MA0100.3	MA0100.3.MYB	4.86183	74	83	accaagtgct
MA0100.3	MA0100.3.MYB	4.84626	879	888	ggcagctgcc
MA0100.3	MA0100.3.MYB	4.84626	879	888	ggcagctgcc
MA0100.3	MA0100.3.MYB	4.70421	1733	1742	gctaactgcg
MA0100.3	MA0100.3.MYB	4.69148	1872	1881	tgcgactggg
MA0100.3	MA0100.3.MYB	4.05066	770	779	cccacctggc
MA0100.3	MA0100.3.MYB	4.01172	307	316	aacaacagtg
MA0100.3	MA0100.3.MYB	3.94554	611	620	agcaacaggc
MA0100.3	MA0100.3.MYB	3.82793	798	807	ggtaactgga
MA0100.3	MA0100.3.MYB	3.77442	148	157	cccaattggc
MA0100.3	MA0100.3.MYB	3.67845	543	552	agcaagtggc
MA0100.3	MA0100.3.MYB	3.6121	2081	2090	cccgacggcc
MA0100.3	MA0100.3.MYB	3.41173	342	351	gcccactgga

Supplementary Table S2. Predicted MYB binding sites within the fragment (-500 to +99) of the ESPN promoter in the hTFtarget databases

TF	Start	Stop	Score	*P*	Matched motif
MYB	1574	1582	10.3187	1.37E-05	CAACGGCCC
MYB	1465	1475	11.1348	1.93E-05	GGGGGCAGTTC
MYB	1467	1475	9.81319	7.31E-05	GAACTGCCC
MYB	1933	1957	28.4079	8.41E-11	CCCACCCCGCCCCGCCCCGCCCCGC
MYB	1938	1962	27.8421	1.51E-10	CCGCCCCCACCCCGCCCCGCCCCGC
MYB	1935	1959	27.0658	3.24E-10	CCCCCACCCCGCCCCGCCCCGCCCC

Supplementary Table S3. The sequences used in the dual-luciferase reporter gene assay

Gene	DNA sequence（5’ to 3’）
MYB	GCCACCATGGCCCGAAGACCCCGGCACAGCATATATAG
	CAGTGACGAGGATGATGAGGACTTTGAGATGTGTGAC
	CATGACTATGATGGGCTGCTTCCCAAGTCTGGAAAGCG
	TCACTTGGGGAAAACAAGGTGGACCCGGGAAGAGGA
	TGAAAAACTGAAGAAGCTGGTGGAACAGAATGGAAC
	AGATGACTGGAAAGTTATTGCCAATTATCTCCCGAATC
	GAACAGATGTGCAGTGCCAGCACCGATGGCAGAAAGT
	ACTAAACCCTGAGCTCATCAAGGGTCCTTGGACCAAA
	GAAGAAGATCAGAGAGTGATAGAGCTTGTACAGAAAT
	ACGGTCCGAAACGTTGGTCTGTTATTGCCAAGCACTTA
	AAGGGGAGAATTGGAAAACAATGTAGGGAGAGGTGG
	CATAACCACTTGAATCCAGAAGTTAAGAAAACCTCCTG
	GACAGAAGAGGAAGACAGAATTATTTACCAGGCACAC
	AAGAGACTGGGGAACAGATGGGCAGAAATCGCAAAG
	CTACTGCCTGGACGAACTGATAATGCTATCAAGAACCA
	CTGGAATTCTACAATGCGTCGGAAGGTCGAACAGGAA
	GGTTATCTGCAGGAGTCTTCAAAAGCCAGCCAGCCAG
	CAGTGGCCACAAGCTTCCAGAAGAACAGTCATTTGAT
	GGGTTTTGCTCAGGCTCCGCCTACAGCTCAACTCCCTG
	CCACTGGCCAGCCCACTGTTAACAACGACTATTCCTAT
	TACCACATTTCTGAAGCACAAAATGTCTCCAGTCATGT
	TCCATACCCTGTAGCGTTACATGTAAATATAGTCAATGT
	CCCTCAGCCAGCTGCCGCAGCCATTCAGAGACACTATA
	ATGATGAAGACCCTGAGAAGGAAAAGCGAATAAAGG
	AATTAGAATTGCTCCTAATGTCAACCGAGAATGAGCTA
	AAAGGACAGCAGGTGCTACCAACACAGAACCACACAT

	GCAGCTACCCCGGGTGGCACAGCACCACCATTGCCGA<br>CCACACCAGACCTCATGGAGACAGTGCACCTGTTTCC<br>TGTTTGGGAGAACACCACTCCACTCCATCTCTGCCAGC<br>GGATCCTGGCTCCCTACCTGAAGAAAGCGCCTCGCCA<br>GCAAGGTGCATGATCGTCCACCAGGGCACCATTCTGG<br>ATAATGTTAAGAACCTCTTAGAATTTGCAGAAACACTC<br>CAATTTATAGATTCTTTCTTAAACACTTCCAGTAACCAT<br>GAAAACTCAGACTTGGAAATGCCTTCTTTAACTTCCAC<br>CCCCCTCATTGGTCACAAATTGACTGTTACAACACCAT<br>TTCATAGAGACCAGACTGTGAAAACTCAAAAGGAAAA<br>TACTGTTTTTAGAACCCCAGCTATCAAAAGGTCAATCT<br>TAGAAAGCTCTCCAAGAACTCCTACACCATTCAAACAT<br>GCACTTGCAGCTCAAGAAATTAAATACGGTCCCCTGAA<br>GATGCTACCTCAGACACCCTCTCATCTAGTAGAAGATC<br>TGCAGGATGTGATCAAACAGGAATCTGATGAATCTGGA<br>ATTGTTGCTGAGTTTCAAGAAAATGGACCACCCTTACT<br>GAAGAAAATCAAACAAGAGGTGGAATCTCCAACTGAT<br>AAATCAGGAAACTTCTTCTGCTCACACCACTGGGAAG<br>GGGACAGTCTGAATACCCAACTGTTCACGCAGACCTC<br>GCCTGTGGCAGATGCACCGAATATTCTTACAAGCTCCG<br>TTTTAATGGCACCAGCATCAGAAGATGAAGACAATGTT<br>CTCAAAGCATTTACAGTACCTAAAAACAGGTCCCTGGC<br>GAGCCCCTTGCAGCCTTGTAGCAGTACCTGGGAACCT<br>GCATCCTGTGGAAAGATGGAGGAGCAGATGACATCTT<br>CCAGTCAAGCTCGTAAATACGTGAATGCATTCTCAGCC<br>CGGACGCTGGTCATGTGA
ESPN<br>promo<br>ter (-	ACTGTGAACTGGGAACAATAGTTGTACCTCTCTCATAG<br>CACCTCTGGATTAAATATCCATGTGAAGCATGGTGAGC<br>ACTTGGTGGGTGGTACAGATTTCATAAAAGTGCTACTG

2000	CTGCCGTGAGCTCCCTAGGGCTGCTGGGGAGGACCCA
to +99)	ATTGGCCCGGTGGACATGGGAGAGCCCGGAGAGGACA
WT	GAAGCCTGGCAGGAAGGGCCTGGCTCACAGCCTGAGT
	TTGAGTCATGTCCCGAAAGGAGTCTGAGAGGTTGGTC
	AGCAGGGGTGGGTGCTCACATGTGGCACTAATGGAGA
	TGTCATTAACAACAGTGGCAGGGGCTCAGGGGCATCT
	GCTCAGCCCACTGGAATAGCAATAAAGCATCACCTAGC
	CCTGGCCCACAAAATCACAAAACCATGTGCTAGATGA
	GCTCGTCCATGACCAAGGTGGCCACCTTAGGAGTGGA
	GAACACAAATTGCAGGGCCTTTTGGAATGGAGGAGGC
	CCAGGGGAGGGGTGCCCAAGACAAGCCATTTTAGCAC
	CTCCTCCCAACAGTACCCCTGCCACTTGCTGGCCTCAC
	GGCACACCTGCCTCTGGGCACCCAGAGGAGGGCAGC
	GCCAGCCGCAGTGAGCCTGTTGCTTTGGCCACCAGCT
	CCAGCAGCGAGGCTGCGAGGAAATGACTCCTCCTTCC
	TTTTGTCTGCAAACCACTGGGCAAGCGTCCCAGATTCC
	AGCGGCCACAGGTCCCATGGAGAGAGTGAGGGCGATC
	TGGCCTGGTGGAAGTCGTTGGAAACCCACCTGGCTAG
	GCACAGCCCAGCAGCTCCAGTTACCTGGGCACCTGGG
	AGCGCGGGAGGCCATCACGCAGGTCGCCACTAGGTGG
	CAGGCCCAGGGAAAGGACCGGAGGCAGCTGCCGAGT
	TTAGCACTATGAGTTTAGCACTATGACTCCCACTGTTCC
	TGGAAGGGGCTTCCTTCATTCTACAGCATTTCCAGTGC
	TGACTGTGCCAGGCTGGGGCTGGGGCGGAGTGAAACC
	TTCAGCCCGGGGTGGGGCCAAGCCCCCACGGGACTCC
	AGAGGGGGTGGGTGTTGGGACAGGAAGGGCACAGGG
	TGCTTCAAGATCGCTGATAGGCCGCCTGCACTCCCACA
	GCCATGTAGCTCCCACCTTCTGTCAGCTTTTCCTCGCG
	TCCTTTGGGCACTAGCCGCTCCCACGGCTGGTGTTCAG

	TGGAGCCTGCAGGGTGGCTTGGCACACATTCGCTCTC<br>CTGCCCGCGCCCTCTGGCACTTTCCTGAGCCCCTGCAG<br>GGGTCTCAGCCCCCTCTCTCTGGATGCCGGCGCTCCCG<br>CGTCTCCCCACAAGCCCTAACCTCCCACCCATCCCTGC<br>CTTCTGTGTGGCTGGAAGCCCCTCCACACCGACAGCG<br>GCTGCGGGCGGCCTGAGGCCATGGCGTTCCCAGCGCA<br>CTAGTGGTTCCCGTCCTGCCTTCCTGCCCTCCCCGCTG<br>GAACCTCTGGGGGCAGTTCTCGGATCTGGAGGGACCC<br>TGGAAGGCAGGGCTCTTTGCAATCTCCGGGGATTTCG<br>ACCCAGAGCCCTTCAGGGACGTGGCAGGGCTGCTCCT<br>GCCTCAGGGCCGTTGTCCTCGTGCTCCTCACCCCGCCT<br>GGAATACCCTTCTCGCCGCTCAAACCCAGCCCCACGG<br>CACCTCCTCAGAGACCTTTCCCTGTCCGCCCACGCGGT<br>CCCGACAATCACTCCCCATCACCTCTGGAATTGCGTCG<br>CCGGCGCCTGGAACCGCAGTTAGCGGGCACTGGGCAG<br>ATGAATGAATTTGTCTGTGCCTGGACGGCTCTCCAATT<br>CGAACCCAGTTTTGCTGCCCTCTGGGGTCTCAACTGTT<br>ACGTGAGGCAAATTAGGAGAGAAGCCCCTGGGCACCT<br>TGCCCCAGTCGCACGAGTGTCCCCGCGTCGCGGCGGG<br>GGCGGGCGGGGAACTCGGGCGGAGGCTGCGGGGCGG<br>GGCGGGGCGGGGTGGGGGCGGGCCCGAGTCTTAAGC<br>CGGCGTCCGCGGGCTCCGGCCCCAGAGCGCGGCGGA<br>GCGGAGCGCCAGGCAGCGCGGAGCGGAGGCCAGGCC<br>CACAGCCGCTCCGCCTCCCGGCCCGCAGATCCCCGAC<br>GGCCGCACCGCGG
ESPN promoter (-1000	GAAACCTTCAGCCCGGGGTGGGGCCAAGCCCCCACGG<br>GACTCCAGAGGGGGTGGGTGTTGGGACAGGAAGGGC<br>ACAGGGTGCTTCAAGATCGCTGATAGGCCGCCTGCAC<br>TCCCACAGCCATGTAGCTCCCACCTTCTGTCAGCTTTT

to +99)	CCTCGCGTCCTTTGGGCACTAGCCGCTCCCACGGCTGG
WT	TGTTCAGTGGAGCCTGCAGGGTGGCTTGGCACACATT
	CGCTCTCCTGCCCGCGCCCTCTGGCACTTTCCTGAGCC
	CCTGCAGGGGTCTCAGCCCCCTCTCTCTGGATGCCGGC
	GCTCCCGCGTCTCCCCACAAGCCCTAACCTCCCACCCA
	TCCCTGCCTTCTGTGTGGCTGGAAGCCCCTCCACACCG
	ACAGCGGCTGCGGGCGGCCTGAGGCCATGGCGTTCCC
	AGCGCACTAGTGGTTCCCGTCCTGCCTTCCTGCCCTCC
	CCGCTGGAACCTCTGGGGGCAGTTCTCGGATCTGGAG
	GGACCCTGGAAGGCAGGGCTCTTTGCAATCTCCGGGG
	ATTTCGACCCAGAGCCCTTCAGGGACGTGGCAGGGCT
	GCTCCTGCCTCAGGGCCGTTGTCCTCGTGCTCCTCACC
	CCGCCTGGAATACCCTTCTCGCCGCTCAAACCCAGCCC
	CACGGCACCTCCTCAGAGACCTTTCCCTGTCCGCCCAC
	GCGGTCCCGACAATCACTCCCCATCACCTCTGGAATTG
	CGTCGCCGGCGCCTGGAACCGCAGTTAGCGGGCACTG
	GGCAGATGAATGAATTTGTCTGTGCCTGGACGGCTCTC
	CAATTCGAACCCAGTTTTGCTGCCCTCTGGGGTCTCAA
	CTGTTACGTGAGGCAAATTAGGAGAGAAGCCCCTGGG
	CACCTTGCCCCAGTCGCACGAGTGTCCCCGCGTCGCG
	GCGGGGGCGGGCGGGGAACTCGGGCGGAGGCTGCGG
	GGCGGGGCGGGGCGGGGTGGGGGCGGGCCCGAGTCT
	TAAGCCGGCGTCCGCGGGCTCCGGCCCCAGAGCGCGG
	CGGAGCGGAGCGCCAGGCAGCGCGGAGCGGAGGCCA
	GGCCCACAGCCGCTCCGCCTCCCGGCCCGCAGATCCC
	CGACGGCCGCACCGCGG
ESPN	CAGGGCTCTTTGCAATCTCCGGGGATTTCGACCCAGAG
promo	CCCTTCAGGGACGTGGCAGGGCTGCTCCTGCCTCAGG
ter (-	GCCGTTGTCCTCGTGCTCCTCACCCCGCCTGGAATACC

500 to +99) WT	CTTCTCGCCGCTCAAACCCAGCCCCACGGCACCTCCTC<br>AGAGACCTTTCCCTGTCCGCCCACGCGGTCCCGACAA<br>TCACTCCCCATCACCTCTGGAATTGCGTCGCCGGCGCC<br>TGGAACCGCAGTTAGCGGGCACTGGGCAGATGAATGA<br>ATTTGTCTGTGCCTGGACGGCTCTCCAATTCGAACCCA<br>GTTTTGCTGCCCTCTGGGGTCTCAACTGTTACGTGAGG<br>CAAATTAGGAGAGAAGCCCCTGGGCACCTTGCCCCAG<br>TCGCACGAGTGTCCCCGCGTCGCGGCGGGGGCGGGCG<br>GGGAACTCGGGCGGAGGCTGCGGGGCGGGGCGGGGC<br>GGGGTGGGGGCGGGCCCGAGTCTTAAGCCGGCGTCCG<br>CGGGCTCCGGCCCCAGAGCGCGGCGGAGCGGAGCGC<br>CAGGCAGCGCGGAGCGGAGGCCAGGCCCACAGCCGC<br>TCCGCCTCCCGGCCCGCAGATCCCCGACGGCCGCACC<br>GCGG
ESPN promoter (-500 to +99) MT1	CAGGGCTCTTTGCAATCTCCGGGGATTTCGACCCAGAG<br>CCCTTCAGGGACGTGGCAGGGCTGCTCCTGCCTCAGG<br>GCCGTTGTCCTCGTGCTCCTCACCCCGCCTGGAATACC<br>CTTCTCGCCGCTCAAACCCAGCCCCACGGCACCTCCTC<br>AGAGACCTTTCCCTGTCCGCCCACGCGGTCCCGACAA<br>TCACTCCCCATCACCTCTGGAATTGCGTCGCCGGCGCC<br>TGGAACCGCAGTTAGCGGGCACTGGGCAGATGAATGA<br>ATTTGTCTGTGCCTGGACGGCTCTCCAATTCGAACCCA<br>GTTTTGCTGCCCTCTGGGGT**AGACCAGTGG**ACGTGAG<br>GCAAATTAGGAGAGAAGCCCCTGGGCACCTTGCCCCA<br>GTCGCACGAGTGTCCCCGCGTCGCGGCGGGGGCGGGC<br>GGGGAACTCGGGCGGAGGCTGCGGGGCGGGGCGGGG<br>CGGGGTGGGGGCGGGCCCGAGTCTTAAGCCGGCGTCC<br>GCGGGCTCCGGCCCCAGAGCGCGGCGGAGCGGAGCG<br>CCAGGCAGCGCGGAGCGGAGGCCAGGCCCACAGCCG

	CTCCGCCTCCCGGCCCGCAGATCCCCGACGGCCGCACCGCGG
ESPN promoter (-500 to +99) MT2	CAGGGCTCTTTGCAATCTCCGGGGATTTCGACCCAGAGCCCTTCAGGGACGTGGCAGGGCTGCTCCTGCCTCAGGGCCGTTGTCCTCGTGCTCCTCACCCCGCCTGGAATACCCTTCTCGCCGCTCAAACCCAGCCCCACGGCACCTCCTCAGAGACCTTTCCCTGTCCGCCCACGCGGTCCCGACAATCACTCCCCATCACCTCTGGAATTGCGTCGCCGGCGCCTGGAACCGCAGTTAGCGGGCACTGGGCAGATGAATGAATTTGTCTGTGCCTGGACGGCTCTCCAATTCGAACCCAGTTTTGCTGCCCTCTGGGGTCTCAACTGTTACGTGAGGCAAATTAGGAGAGAAGCCCCTGGGCACCTTGCCCCAGTCGCACGAGT**TGAAAATATGATATTATTTTTATTTATTTTCCAGATTTATTCTTAGTATTTTATTTTATTTTATTTTGTTTTTATTTAAATC**GTCTTAAGCCGGCGTCCGCGGGCTCCGGCCCCAGAGCGCGGCGGAGCGGAGCGCCAGGCAGCGCGGAGCGGAGGCCAGGCCCACAGCCGCTCCGCCTCCCGGCCCGCAGATCCCCGACGGCCGCACCGCGG
ESPN promoter (-500 to +99) MT3	CAGGGCTCTTTGCAATCTCCGGGGATTTCGACCCAGAGCCCTTCAGGGACGTGGCAGGGCTGCTCCTGCCTCAGGGCCGTTGTCCTCGTGCTCCTCACCCCGCCTGGAATACCCTTCTCGCCGCTCAAACCCAGCCCCACGGCACCTCCTCAGAGACCTTTCCCTGTCCGCCCACGCGGTCCCGACAATCACTCCCCATCACCTCTGGAATTGCGTCGCCGGCGCCTGGAACCGCAGTTAGCGGGCACTGGGCAGATGAATGAATTTGTCTGTGCCTGGACGGCTCTCCAATTCGAACCCAGTTTTGCTGCCCTCTGGGGT**AGACCAGTGG**ACGTGAGGCAAATTAGGAGAGAAGCCCCTGGGCACCTTGCCCCA

GTCGCACGAGT**<u>TGAAAATATGATATTATTTTTATTTAT</u>**

<u>TTTCCAGATTTATTCTTAGTATTTTATTTTATTTTATT</u>

<u>TTGTTTTTATTTAAATC</u>GTCTTAAGCCGGCGTCCGCG

GGCTCCGGCCCCAGAGCGCGGCGGAGCGGAGCGCCA

GGCAGCGCGGAGCGGAGGCCAGGCCCACAGCCGCTC

CGCCTCCCGGCCCGCAGATCCCCGACGGCCGCACCGC

GG

Supplementary Table S4. The probe sequences used for electrophoretic mobility shift assay.

Gene	DNA sequence（5’ to 3’）
ESPN-wt	CCCAGTCGCACGAGTGTCCCCGCGTCGCGGCGGGGGCGGGCGGGGAACTCGGGCGGAGGCTGCGGGGCGGGGCGGGGCGGGGTGGGGGCGGGCCCGAGTCTTAAGCCG
ESPN-mut	CCCAGTCGCACGAGT**TGAAAATATGATATTATTTTTATTTATTTTCCAGATTTATTCTTAGTATTTTATTTTATTTTATTTTGTTTTTATTTAAATC**GTCTTAAGCCG

Supplementary Figure - original blot/gel images

Figure 3D

AMC-HN-8

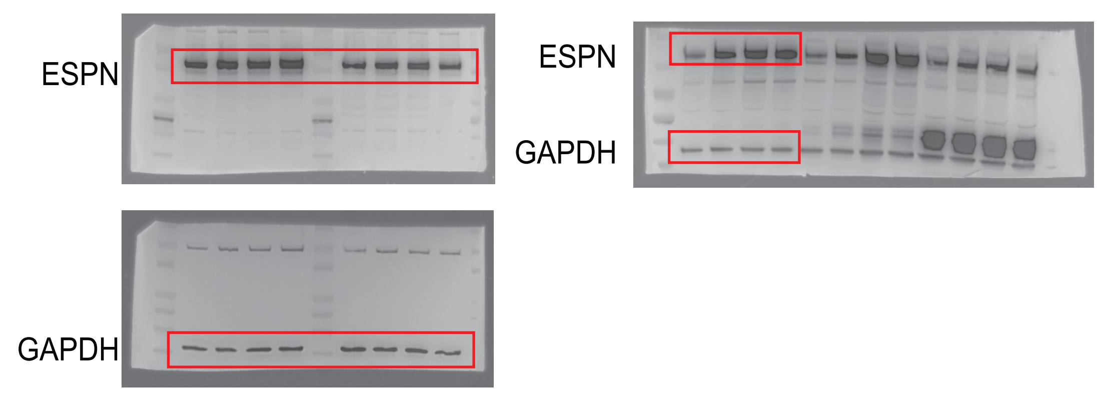

Fadu

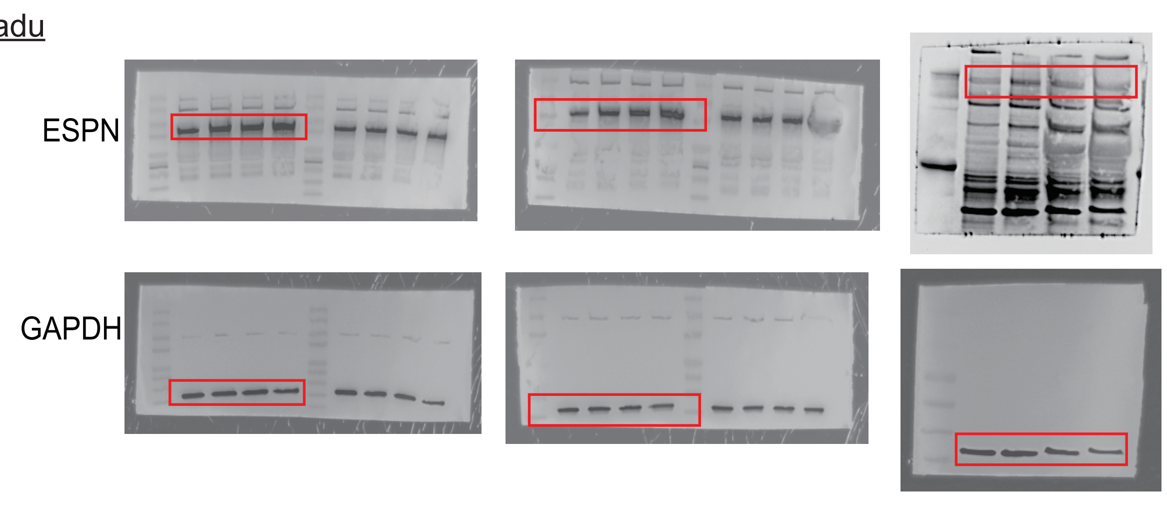

Figure 4C

AMC-HN-8

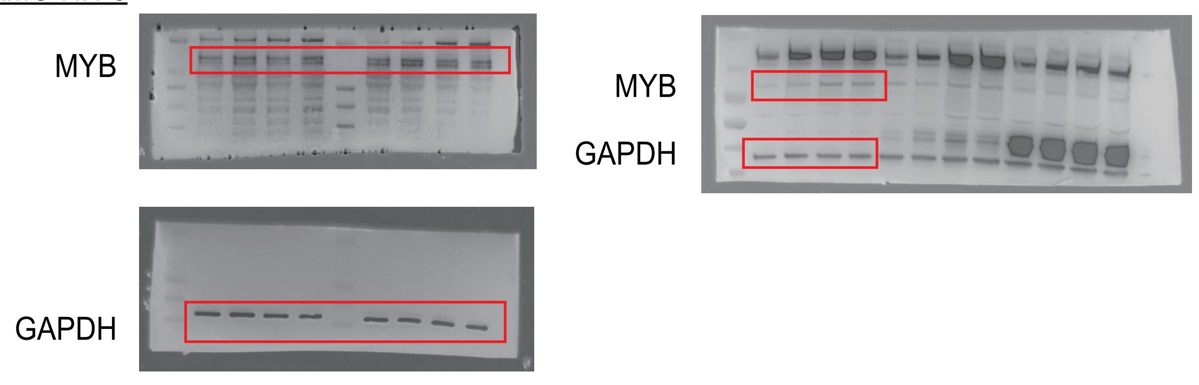

Fadu

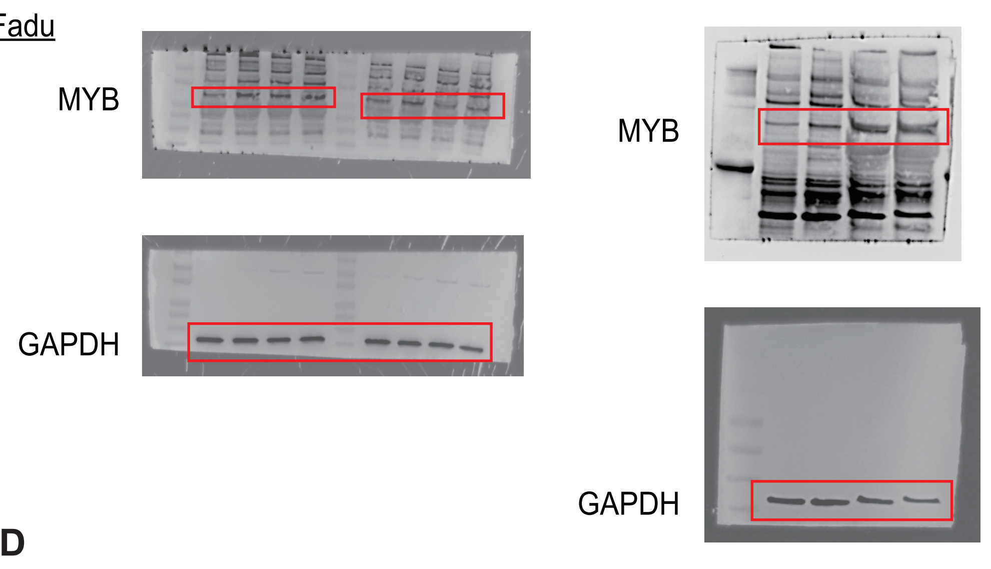

Fig 4D

AMC-HN-8

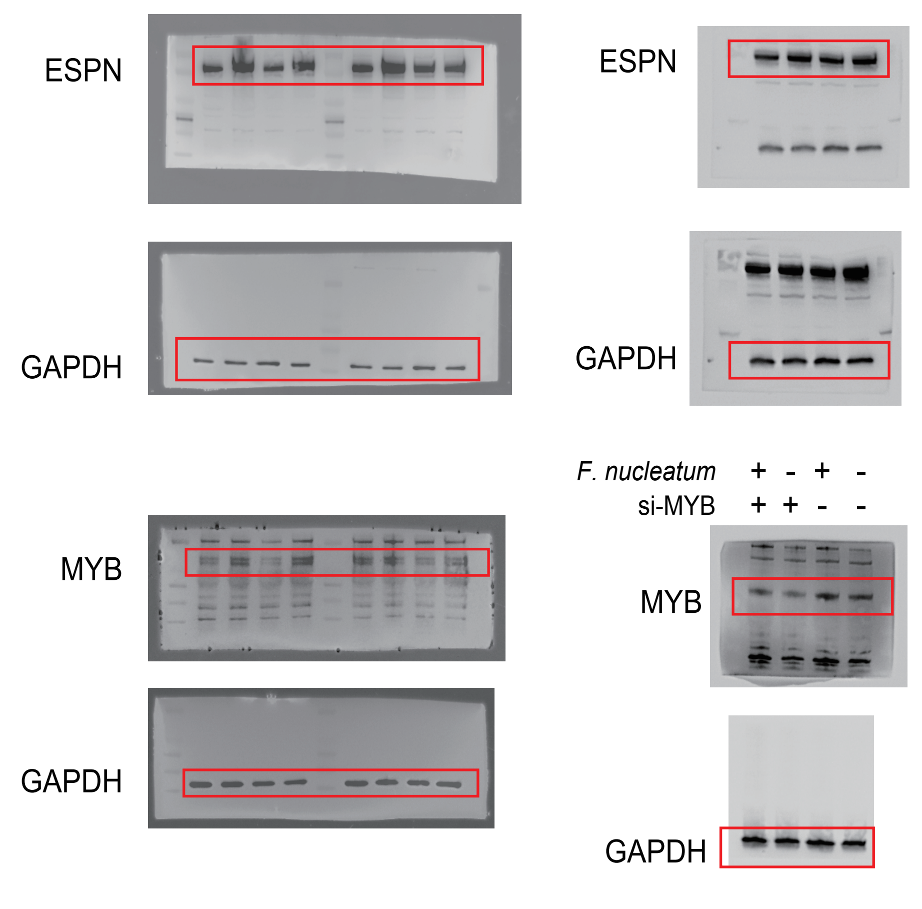

FaDu

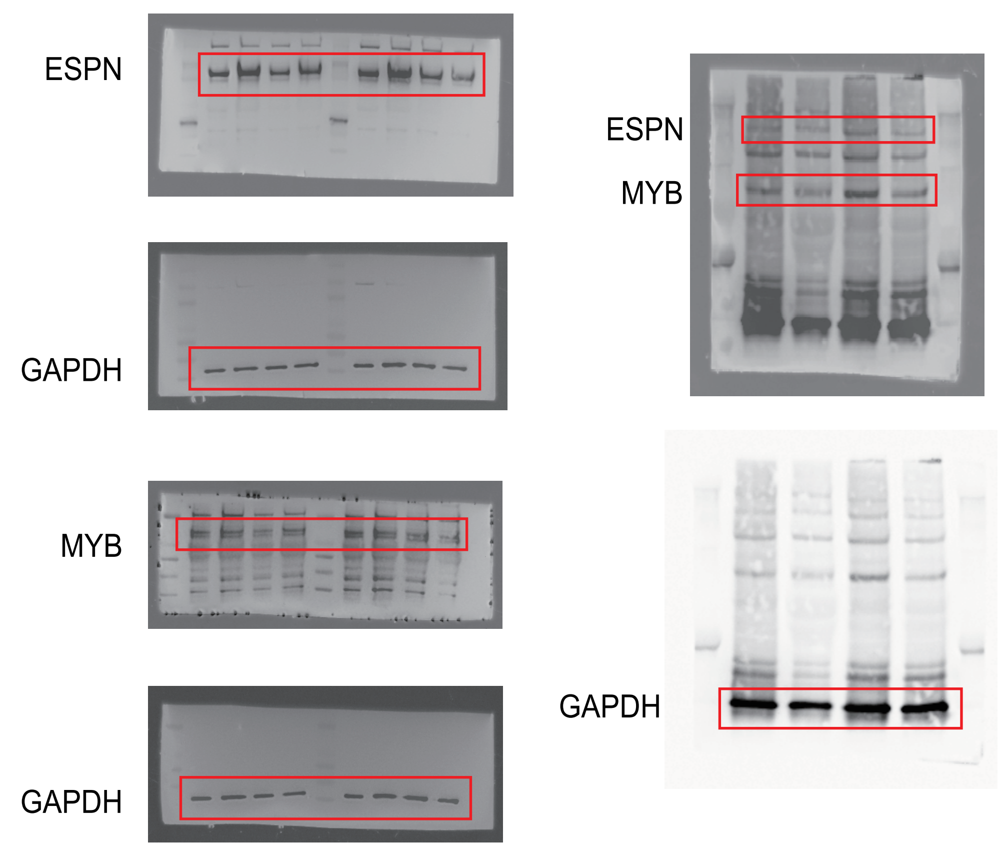

Fig 4G

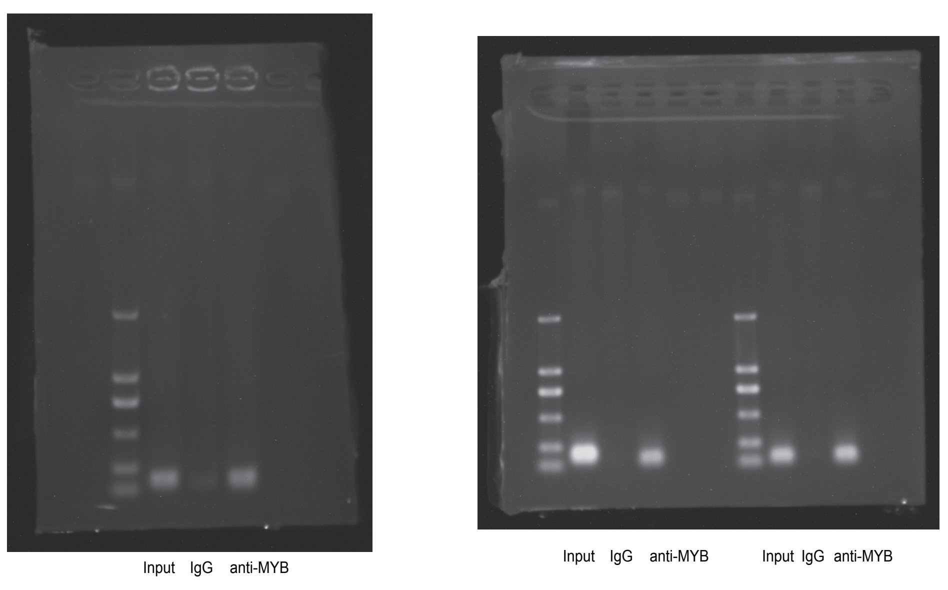

Fig 4H

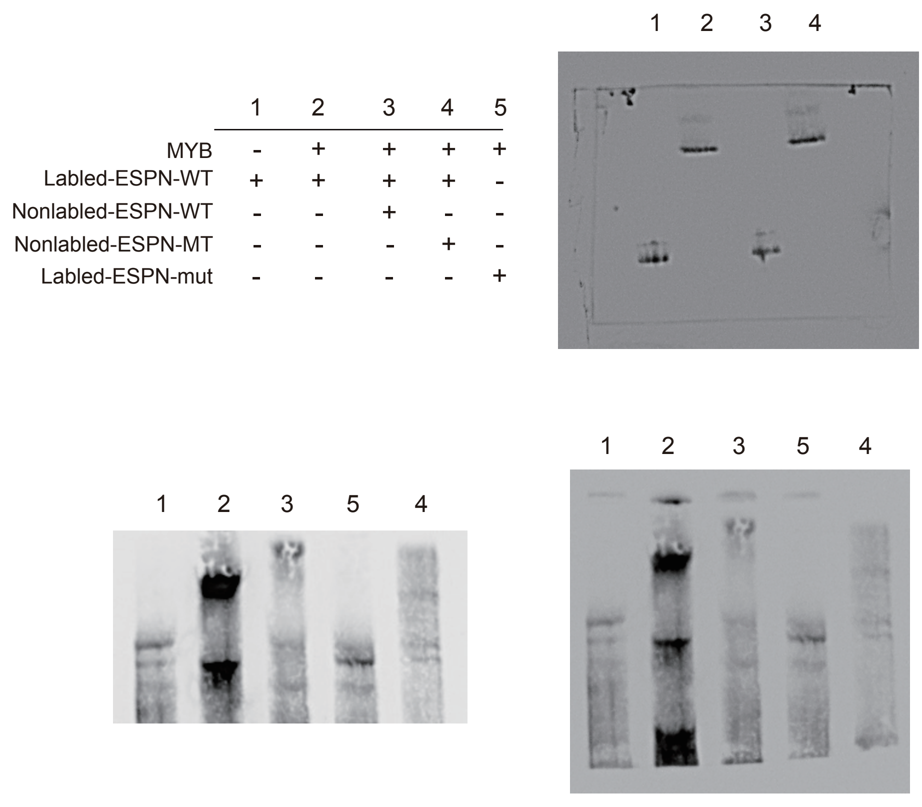

Fig 4I

AMC-HN-8 FaDu

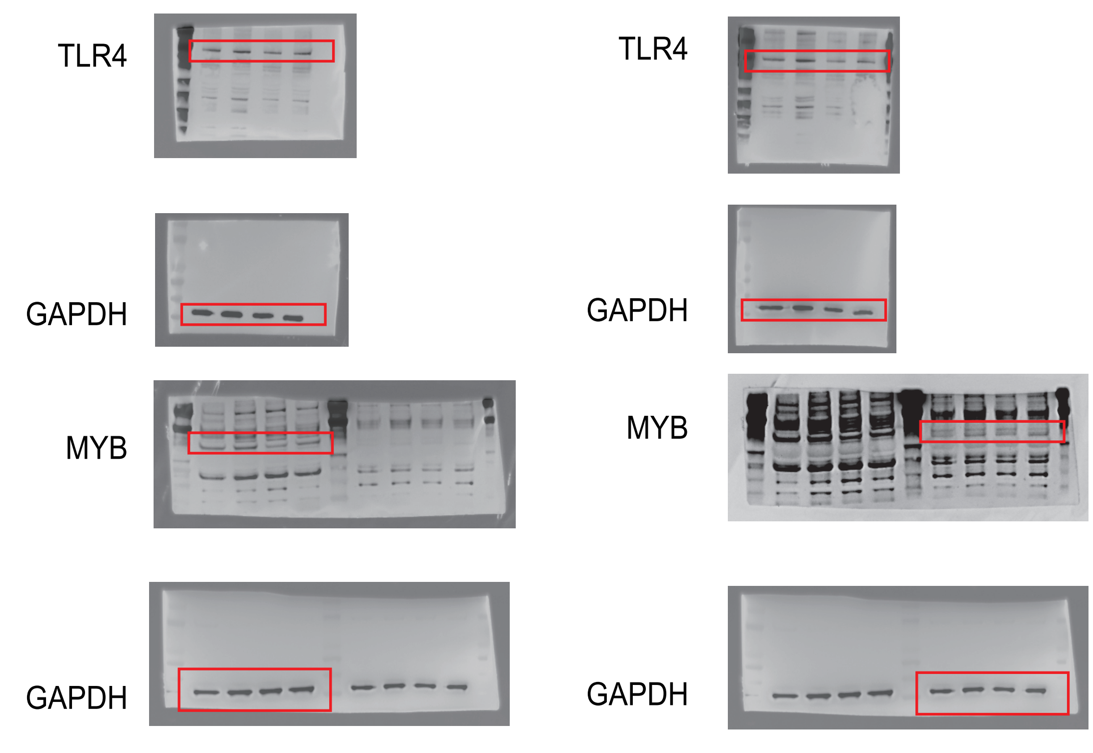

Supplement: Supplementary file 2 — Supplementary Information [file 42003_2026_9913_MOESM2_ESM.pdf]
