# Supplementary material for: Fusobacterium nucleatum promotes tumor extravasation and metastasis in head and neck cancer via TLR4/MYB/ESPN axis
Source: Commun Biol. 2026 Mar 25;9:664. doi: 10.1038/s42003-026-09913-3 (PMC13181126; doi:10.1038/s42003-026-09913-3)
Supplement: Supplementary file 3 — Description of Additional Supplementary Files [file 42003_2026_9913_MOESM3_ESM.pdf]

Description of Additional Supplementary Files

File name: Supplementary Data 1

Description: The source data can be found in Supplementary Data 1

File name: Supplementary Data 2

Description: To elucidate the regulatory mechanism of F. nucleatum on ESPN gene expression, transcription factors predicted to bind to the ESPN promoter region were identified using the JASPAR, CIS-BP, hTFtarget, and HOCOMOCO databases
