# Supplementary material for: Fusobacterium nucleatum promotes tumor extravasation and metastasis in head and neck cancer via TLR4/MYB/ESPN axis
Source: Commun Biol. 2026 Mar 25;9:664. doi: 10.1038/s42003-026-09913-3 (PMC13181126; doi:10.1038/s42003-026-09913-3)
Supplement: Supplementary file 6 — Reporting summary [file 42003_2026_9913_MOESM6_ESM.pdf]

nature portfolio

Corresponding author(s): Hongli Gong, Chi-Yao Hsueh, Liang Zhou

Last updated by author(s): Feb 6, 2026

Reporting Summary

Nature Portfolio wishes to improve the reproducibility of the work that we publish. This form provides structure for consistency and transparency in reporting. For further information on Nature Portfolio policies, see our Editorial Policies and the Editorial Policy Checklist.

Statistics

For all statistical analyses, confirm that the following items are present in the figure legend, table legend, main text, or Methods section.

n/a	Confirmed	
☐	☒	The exact sample size (*n*) for each experimental group/condition, given as a discrete number and unit of measurement
☐	☒	A statement on whether measurements were taken from distinct samples or whether the same sample was measured repeatedly
☐	☒	The statistical test(s) used AND whether they are one- or two-sided<br>*Only common tests should be described solely by name; describe more complex techniques in the Methods section.*
☐	☒	A description of all covariates tested
☐	☒	A description of any assumptions or corrections, such as tests of normality and adjustment for multiple comparisons
☐	☒	A full description of the statistical parameters including central tendency (e.g. means) or other basic estimates (e.g. regression coefficient) AND variation (e.g. standard deviation) or associated estimates of uncertainty (e.g. confidence intervals)
☒	☐	For null hypothesis testing, the test statistic (e.g. *F*, *t*, *r*) with confidence intervals, effect sizes, degrees of freedom and *P* value noted<br>*Give P values as exact values whenever suitable.*
☒	☐	For Bayesian analysis, information on the choice of priors and Markov chain Monte Carlo settings
☒	☐	For hierarchical and complex designs, identification of the appropriate level for tests and full reporting of outcomes
☒	☐	Estimates of effect sizes (e.g. Cohen's *d*, Pearson's *r*), indicating how they were calculated

Our web collection on statistics for biologists contains articles on many of the points above.

Software and code

Policy information about availability of computer code

Data collection	The mRNA expression profiling data for 520 primary tumors from the TCGA-HNSC project were obtained from TCGA database (https://portal.gdc.cancer.gov/). Gene expression profiles of GSE 65858 and GSE 117973 were obtained from the Gene Expression Omnibus (GEO) database (http://www.ncbi.nlm.nih.gov/geo/). F. nucleatum abundance data in the public database was obtained from the TCMA database (https://doi.org/10.7924/r4bk1j35s). JASPAR, CIS-BP, hTFtarget, and HOCOMOCO databases were used to predict transcription factors.
Data analysis	Image J (v1.53a), Graphpad prism 10.0, R version 4.5.1, PASS 15.0.5

For manuscripts utilizing custom algorithms or software that are central to the research but not yet described in published literature, software must be made available to editors and reviewers. We strongly encourage code deposition in a community repository (e.g. GitHub). See the Nature Portfolio guidelines for submitting code & software for further information.

Data

Policy information about availability of data

All manuscripts must include a data availability statement. This statement should provide the following information, where applicable:

- Accession codes, unique identifiers, or web links for publicly available datasets
- A description of any restrictions on data availability
- For clinical datasets or third party data, please ensure that the statement adheres to our policy

The source data can be found in Supplementary Data. The transcriptome sequencing data is available in the NCBI GEO repository (https://www.ncbi.nlm.nih.gov/geo/) under accession number GSE236237 (DOI: 10.1099/mgen.0.001221).

Research involving human participants, their data, or biological material

Policy information about studies with human participants or human data. See also policy information about sex, gender (identity/presentation), and sexual orientation and race, ethnicity and racism.

Reporting on sex and gender	Gender was included as a covariate.
Reporting on race, ethnicity, or other socially relevant groupings	not applicable.
Population characteristics	Age, gender, smoking history, drinking history, TNM stage.
Recruitment	Tissue samples were collected from the Eye & ENT Hospital, Fudan University. All participants provided written informed consent.
Ethics oversight	All experiments and methods were performed in accordance with relevant guidelines and regulations. The study was approved by the Ethics Committee of the Eye & ENT Hospital, Fudan University, China (approval number: 2022076). All participants signed informed consent.

Note that full information on the approval of the study protocol must also be provided in the manuscript.

Field-specific reporting

Please select the one below that is the best fit for your research. If you are not sure, read the appropriate sections before making your selection.

☒ Life sciences ☐ Behavioural & social sciences ☐ Ecological, evolutionary & environmental sciences

For a reference copy of the document with all sections, see nature.com/documents/nr-reporting-summary-flat.pdf

Life sciences study design

All studies must disclose on these points even when the disclosure is negative.

Sample size	A TMA was constructed using fresh HNSCC tumor and paired adjacent normal tissues, testing a total of 77 primary HNSCC tissues collected (Cohort 1, Table 1). Additionally, 104 FFPE HNSCC samples (Cohort 2, Table 1) were collected for genomic DNA extraction while 78 FFPE HNSCC samples (Cohort 3, Table 1) were used for IHC analysis.
Data exclusions	No data were excluded.
Replication	All experiments were performed at least three times.
Randomization	Mice were allocated to experimental and control groups using a simple randomization process.
Blinding	The data analysis was performed blind to the group allocation.

Reporting for specific materials, systems and methods

We require information from authors about some types of materials, experimental systems and methods used in many studies. Here, indicate whether each material, system or method listed is relevant to your study. If you are not sure if a list item applies to your research, read the appropriate section before selecting a response.

Materials & experimental systems

n/a	Involved in the study
☐	☒ Antibodies
☐	☒ Eukaryotic cell lines
☒	☐ Palaeontology and archaeology
☐	☒ Animals and other organisms
☐	☒ Clinical data
☒	☐ Dual use research of concern
☒	☐ Plants

Methods

n/a	Involved in the study
☒	☐ ChIP-seq
☒	☐ Flow cytometry
☒	☐ MRI-based neuroimaging

Antibodies

Antibodies used	ESPN (#A15908, Abclonal, RRID: AB_2763339; #NBP2-55817, Novus Biologicals, RRID: AB_3341008), MYB (#17800-1-AP, Proteintech, RRID: AB_2148029), TLR4 (#19811-1-AP, Proteintech, RRID: AB_10638446), and GAPDH (#60004-1-Ig, Proteintech, RRID:AB_2107436), Alexa Fluor 647 (#ab150083, Abcam, RRID: AB_2714032), ActinRed 555 ReadyProbes Reagent (#R37112, ThermoFisher).
Validation	All the antibodies were verified by the manufacturers.

Eukaryotic cell lines

Policy information about cell lines and Sex and Gender in Research

Cell line source(s)	The human HNSCC cell line AMC-HN-8 (RRID: CVCL_5966) was provided by Professor S. Y. Kim, and the human HNSCC cell line FaDu (RRID: CVCL_1218) was purchased from the Cell Bank of Type Culture Collection of Chinese Academy of Sciences (CBTCCCAS).
Authentication	The human HNSCC cell line AMC-HN-8 (RRID: CVCL_5966) was provided by Professor S. Y. Kim, and the human HNSCC cell line FaDu (RRID: CVCL_1218) was purchased from the Cell Bank of Type Culture Collection of Chinese Academy of Sciences (CBTCCCAS).
Mycoplasma contamination	We confirm all cell lines were not contaminated with mycoplasma.
Commonly misidentified lines (See ICLAC register)	not applicable.

Animals and other research organisms

Policy information about studies involving animals; ARRIVE guidelines recommended for reporting animal research, and Sex and Gender in Research

Laboratory animals	BALB/c-nu/nu mice
Wild animals	The male, 4-5 weeks old BALB/c-nu/nu mice were purchased from Shanghai Zhanluan Biotechnology Co. Ltd (China).
Reporting on sex	male
Field-collected samples	not applicable.
Ethics oversight	The animal experiment protocol was approved by the Ethics Committee of the Eye & ENT Hospital, Fudan University.

Note that full information on the approval of the study protocol must also be provided in the manuscript.

Clinical data

Policy information about clinical studies

All manuscripts should comply with the ICMJE guidelines for publication of clinical research and a completed CONSORT checklist must be included with all submissions.

Clinical trial registration	not applicable.
Study protocol	Retrospective clinical study
Data collection	Clinicopathological characteristics were retrospectively collected using inpatient records. Archived FFPE tumor tissues were collected. Sections (10 μm thick) were prepared from FFPE tumour tissue samples.

Outcomes	Disease-free survival (DFS) was defined as survival time from the end of treatment to recurrence (local, regional), distant metastasis or death.

Plants

Seed stocks	not applicable.
Novel plant genotypes	not applicable.
Authentication	not applicable.
